# HER-2-Targeted Electrochemical Sensors for Breast Cancer Diagnosis: Basic Principles, Recent Advancements, and Challenges

**DOI:** 10.3390/bios15040210

**Published:** 2025-03-25

**Authors:** Leila Kudreyeva, Fatima Kanysh, Aliya Sarsenbayeva, Moldir Abu, Duisek Kamysbayev, Kamilya Kedelbayeva

**Affiliations:** 1 Department of Analytical Chemistry, Colloidal Chemistry and Technology of Rare Elements, Faculty of Chemistry and Chemical Technology, Al-Farabi Kazakh National University, Almaty 050040, Kazakhstan; fatima_q.m_02@mail.ru (F.K.); a.moldir.b@mail.ru (M.A.); duisek.kamysbayev@kaznu.kz (D.K.); 2 Department of Cardiology Asfendiyarov, Kazakh National Medical University, Almaty 050012, Kazakhstan; kamilla-km@mail.ru

**Keywords:** breast cancer, biomarker, biosensor, electrochemical sensor, interfering agent

## Abstract

In this literature review, methods for the detection of breast cancer biomarkers and the operation of electrochemical sensors are considered. The work of sensors in the determination of breast cancer biomarkers was systematized, a comparative table with other methods was compiled, as was a classification of sensors depending on their intended use. The various traditional methods for the diagnosis of breast cancer biomarkers are described, including mammography, ultrasound, magnetic resonance imaging, positron emission computed tomography, computed tomography, single-photon emission computed tomography, and biopsy, and their advantages and disadvantages are presented. Key sensor parameters for the detection of breast cancer biomarkers are compared, such as the detection limit, linear detection range, response time, sensitivity, and other characteristics depending on the analyte being analyzed. Based on the reviewed scientific papers, the significance of electrochemical sensors in detecting the biomarkers of breast cancer is demonstrated. The types of tumor biomarkers identified by biosensors were analyzed, with a particular focus on HER2. Studies on HER2 detection using electrochemical methods are compared and systematized, and the features of electrochemical biosensors for determining this biomarker are characterized. Possible interfering agents affecting the accuracy of HER2 determination under experimental conditions are considered, their mechanisms of action are analyzed, and ways to eliminate them are proposed. This report provides a summary of the current aspects of scientific research on electrochemical sensors for the detection of breast cancer biomarkers. The development of electrochemical biosensors opens up new prospects for the early diagnosis and prognosis of breast cancer treatment.

## 1. Introduction

According to the World Health Organization (WHO), cancer will be the leading cause of approximately 12 million deaths by 2030 [1]. According to the updated estimates from the International Agency for Research on Cancer (IARC), the global cancer incidence situation was a major concern in 2022.

**Figure 1:**
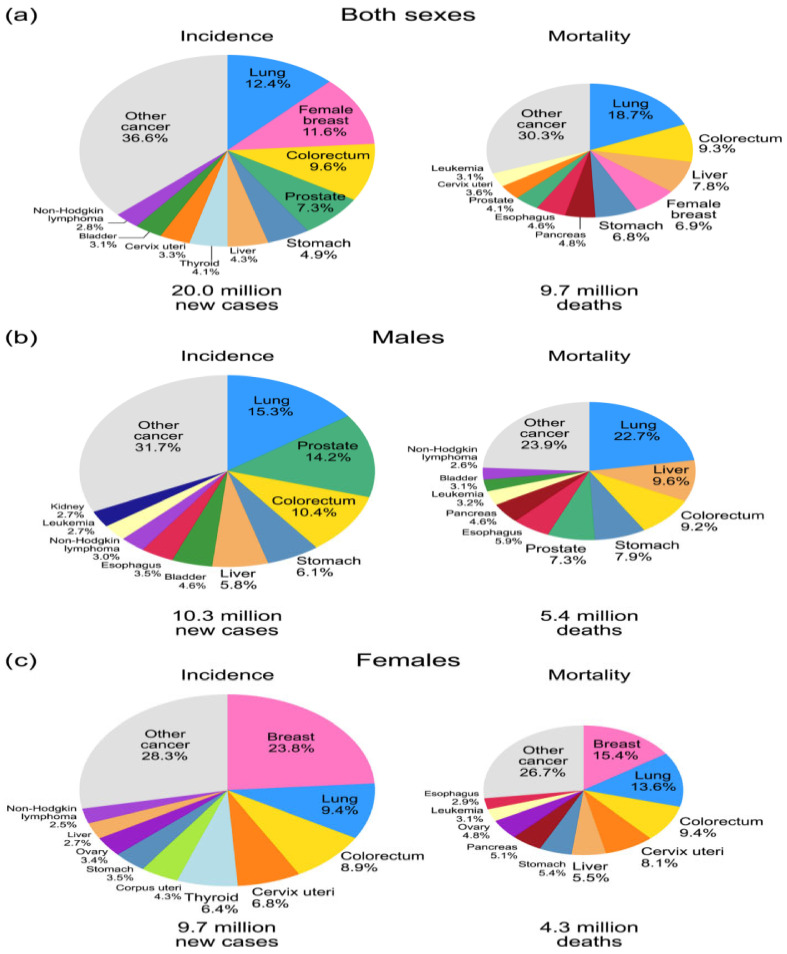
Distribution of cancer morbidity and mortality among men and women. The data are presented for both sexes (**a**), males (**b**), and females (**c**) [2].

According to the latest statistics (Figure 1), the total number of cancer cases and deaths worldwide was about 20 million new cases and 9.7 million deaths. Among women, breast cancer is the most diagnosed cancer (11.6%) and one of the leading causes of death. In addition, colorectal cancer, prostate cancer, and stomach cancer are also common [2].

The figure shows pie charts showing the incidence of various types of malignant neoplasms and the mortality rate among the population. The data are reflected for both sexes (a), men (b), and women (c). In women, breast cancer occupies a leading position among all types of malignant tumors (23.8% of cases), which underlines its epidemiological significance and the need to develop effective diagnostic methods.

In recent years, the prevalence of cancer in Kazakhstan has reached an alarming level. By the end of 2023, the total number of reported cancer cases in the entire population exceeded 205,000. This indicator highlights the need for systemic cancer control measures.

**Figure 2:**
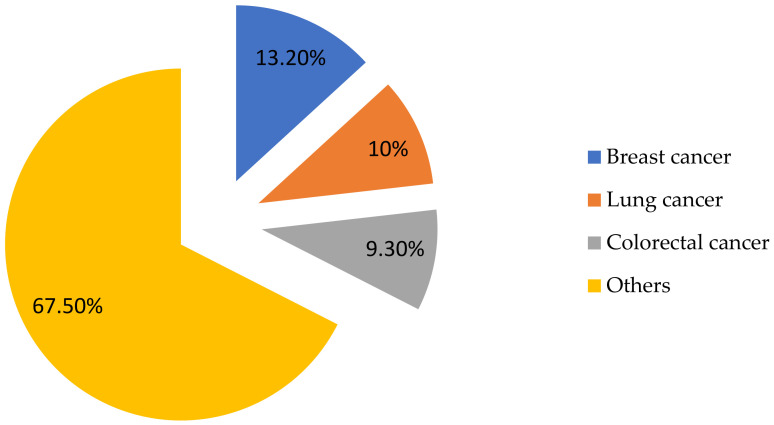
Cancer incidence statistics in Kazakhstan in 2023 [3].

In this context, a special place is occupied by breast cancer, which accounts for 13.2% of all cases and remains the most common cancer among women. Lung cancer accounts for 10% and colorectal cancer for 9.3%, while other cancers account for 67.5% of all cases (Figure 2). A particularly high incidence of breast cancer requires strengthening measures for its early diagnosis and prevention [3].

The diagram illustrates the distribution of cancer cases among the population of Kazakhstan. Other cancers account for the largest share (67.5%), but breast cancer (13.2%) is one of the most common, second only to lung cancer (10%) and colorectal cancer (9.3%). This fact indicates a high need for the early diagnosis and monitoring of this disease, which makes the development of electrochemical biosensors for the detection of appropriate biomarkers extremely relevant.

Cancer usually occurs as a result of certain genetic mutations in normal cells [4]. It is characterized by the abnormal growth and development of cells outside of their natural boundaries [5]. There are more than 200 types of cancers that can affect more than 60 organs in the human body. It is estimated that at least 90% of cancer-related deaths are due to primary tumor metastasis [6].

**Figure 3:**
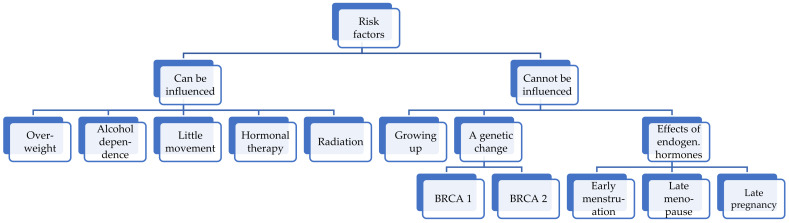
Breast cancer risk factors.

Breast cancer (BC) is a malignant neoplasm that originates from the epithelial cells of breast tissue [7]. The development of breast cancer does not have a single etiological factor. In 3–10% of patients with this disease, its occurrence is associated with mutations in the BRCA1, BRCA2, CHEK, NBS1, and TP53 genes. This pathology is characterized by the presence of risk factors [8], which are shown in Figure 3.

Modifiable and unmodifiable risk factors are schematically presented. The first group includes overweight, alcohol dependence, low physical activity, hormone therapy, and radiation exposure. The second group includes age-related changes, genetic predisposition (BRCA1 and BRCA2 mutations), as well as endogenous hormonal factors such as early menstruation, late menopause, and late pregnancy. The identification of these risk factors in combination with the biomarkers of the malignant process will improve the accuracy of early diagnosis and reduce the mortality rate of breast cancer.

The development of breast cancer is influenced by both modifiable and non-modifiable risk factors, as shown in the diagram. While genetic mutations and hormonal changes cannot be controlled, lifestyle factors such as physical activity, diet, and alcohol consumption play a crucial role in reducing the risk. Understanding these factors is essential for early prevention and targeted therapeutic strategies.

Globally, cancer is a major financial burden and is becoming more prevalent in many nations. Nonetheless, the early diagnosis of breast cancer can lead to the development of control strategies, a decrease in the incidence of breast cancer, and an increase in survival rates [9]. Recently, there has been a development of biosensors that incorporate nanomaterials, showcasing highly selective surfaces for biological or chemical recognition [10]. The integration of nanomaterials boosts sensor sensitivity by increasing the surface area and promoting electron transmission, thereby enhancing the performance of the analytical device [11]. Among these nanomaterials, two-dimensional nanomaterials are characterized by their nanosheet or layered multi-sheet structures, which markedly affect the electrochemical properties of the sensor [12].

## 2. Breast Cancer Diagnostic Methods

There are several methods that can be utilized for the diagnosis of breast cancer, including mammography, biopsy, enzyme immunoassay (ELISA), liquid biopsy, immunohistochemistry (IHC), fluorescent in situ hybridization (FISH), and others [13]. These expensive methods have low sensitivity, often give false results, and can cause mutations in tissues due to exposure to X-rays. Therefore, the urgent task is to find cost-effective, simple, and fast diagnostic methods. The use of various methods for the detection of breast cancer allows you to visualize the morphology and localization of tumor tissue, providing doctors with valuable clinical information. The main diagnostic methods include mammography, ultrasound, magnetic resonance imaging (MRI), positron emission tomography (PET), computed tomography (CT), and single-photon emission computed tomography (SPECT) [14]. Detailed information on the characteristics and disadvantages of these methods is presented in Table 1.

Mammography is the main method of studying the mammary glands, and its effectiveness has been proven: regular use reduces mortality of breast cancer (BC) by 30–40%. However, this method has limitations, including a high rate of false-positive and false-negative results. Radiologists still do not detect 10–30% of cancer cases. In addition, 80% of repeated examinations after detecting suspicious changes are normal, and 40% of biopsies performed are associated with benign formations [23].

These methods are used to diagnose breast cancer in accordance with the TNM (Tumor–Node–Metastasis) system, which takes into account the size of the tumor (Tumor), the degree of its spread to the lymph nodes (Node) and the presence of metastases (Metastasis). These factors have an important prognostic value in the diagnosis of cancer. However, it is not sufficient to rely solely on the TNM system to prescribe an individualized treatment regimen. In this regard, the research and development of biomarkers that determine the molecular characteristics of breast cancer are relevant today [24].

Moreover, the development of novel, easy-to-use, and reasonably priced platforms—like novelty electrochemical sensors—offers exceptional sensitivity, which is crucial for the early diagnosis of cancer. Clinical cancer diagnostics will have a significant demand for it. Electrochemical impedance spectroscopy (EIS), square-wave voltammetry (SWV), differential pulse voltammetry (DPV), cyclic voltammetry (CV), and others are the primary methods used to obtain electrochemical measurements [25]. Chromatographic techniques can be largely replaced by quick and precise electrochemical techniques that use an inexpensive apparatus with various customized electrodes. For instance, in a comparison of electrochemical biosensors with traditional methods, such as mammography or biopsy, the first one is economically efficient and affordable. Fast response, high sensitivity, the ease of instrument preparation, scalability, and relatively low cost make electrochemical biosensors a promising detection technique. The identification of the tumor and its type (cancerous or benign) is made possible by the precise quantification of over/underexpressed biomarkers. Given these characteristics, electrochemistry is a better technique for early cancer biomarker detection than time-consuming and low-sensitivity analytical techniques like surface plasmon resonance and bioluminescence analysis [26]. For example, in the application of biopsy, there is a likelihood of no tumor cells, unnecessary surgery, it may cause tumor metastasis, be performed at a late stage, and expensive [21].

### 2.1. Commercializing Electrochemical Biosensors for Clinical Use

The electrochemical biosensors market is anticipated to reach a valuation of USD 24 million and increase at a rate of 9.7% each year [27]. The electrochemical biosensors will be valued at USD 33 billion and are utilized in clinical applications for point-of-care (POC) testing. In the future, new species will be able to utilize this increasingly sophisticated use of electrochemical biosensors, which are effective in detecting biowarfare agents. In this sense, there is a great deal of room for technological and design changes. The unfortunate fact that glucose test strips are the only product of the electrochemical biosensor industry to have achieved commercial success could be changed by combining electrochemical biosensing with nanotechnology and meeting the growing demand for low-cost, mass-produced, single-use biosensors.

Machine learning has been applied to many different applications and aids in the interpretation of massive sensing data. The collected data occasionally contain noise and disturbances and are not always clear. Such data can be analyzed with machine learning to provide the desired outcomes. Numerous medical difficulties will be addressed by this technology. By monitoring rapid, highly localized, and transient changes in the tumor’s response to chemotherapy and radiation therapy, biosensors will aid in the development of personalized cancer treatment. There will be several chances for the design and development of biosensors that use mechanisms based on nanomaterials to solve a variety of intricate medical issues [28].

Today, glucose sensors continue to control 85% of the biosensor market. Biosensors for various biomarkers are not yet widely commercially translated. Finding technologies that cater to sizable enough commercial markets, such as the glucose monitoring market, to warrant the high research and regulatory expenses of developing a new point-of-care diagnostic gadget is one aspect of this issue. Even though there is a sizable market potential, investing in diagnostic technologies is made riskier by the technical difficulties in bringing biosensing devices from the lab to the market, which lengthen the development period and raise the cost of technology translation.

The development of novel ultrasensitive signal transduction systems to increase the limit of detection (LOD) of biosensors is a major interest of the analytical chemistry community; nonetheless, many of these techniques are created in clean buffers and tested on purified targets. Despite their frequent creativity and elegance, these assays frequently cannot be modified to operate with clinical samples or procedures [29]. Therefore, the main task is to find and realize stable, sensitive, cost-effective point-of-care biosensors in various sectors of life from laboratory analysis to the commercialization industry.

### 2.2. Biomarkers for Breast Cancer Diagnosis

According to the definition from the International Cancer Institute, a biomarker is a biological substance present in biological matrices or tissues [30]. Cancer is often associated with biomarkers found in or released by the tumor cells themselves. A deviation in the level of these biomarkers from the norm may indicate the presence of cancer. The use of breast cancer (BC) biomarkers in healthcare depends on the ability to identify multiple tumor markers with high selectivity and sensitivity. In oncology, biomarkers are any measurable indicators that indicate the presence of cancer in the body, the possible transition of a benign process to a malignant one, and they predict the development of a tumor or its response to treatment [31]. The rapid growth in the number of studies devoted to the role of biomarkers in oncology is due to their ability to quickly identify the mechanisms of the proliferation and transformation of tumor cells [32]. All biomarkers of breast cancer (BC) are divided into two large groups [33]:

- Proteomic biomarkers: RS/DJ-1, heat shock proteins 60 (HSP60) and 90 (HSP90), mucin 1 (MUC1), carbohydrate antigen 15-3 (CA15-3), and carbohydrate antigen 27-29 (CA27-29).
- Gene biomarkers: breast cancer-associated BRCA1 and BRCA2 genes, p53 gene, and miRNAs.

Currently, the key prognostic biomarkers of breast cancer are as follows: the estrogen receptor (ER+), which is more often evaluated together with the progesterone receptor (PR+), the human epidermal growth receptor 2 (HER2+), and the androgen receptor (AR), which is used in the classification of breast cancer [34].

The estrogen receptor (ER), progesterone receptor (PR), and human epidermal growth factor receptor (HER2) are most commonly used to detect breast tumors. Currently, new biological markers are being studied to improve diagnostics and treatment methods [35].

The estrogen receptor (ER) is a specific protein that interacts with estrogen. These receptors can be located in the cell nucleus, cytoplasm, or on the membrane. Estrogen, penetrating the nucleus, binds to ER and regulates the work of genes [36,37]. The determination of ER makes it possible to assess the effectiveness of hormone therapy: in patients with ER-positive tumors, hormone therapy slows down tumor growth, while in ER-negative patients, it has no effect [38,39,40].

The progesterone receptor (PR) is a marker of estrogen receptor activity. When PR interacts with chromatin, the activity of genes in the cell changes, which leads to a halt in cell proliferation and triggers mechanisms of self-destruction or changes in the functional state of the cell [37]. In 65–70% of patients, the PR test gives a positive result. With a high level of PR, as a rule, the ER is also positive. If the PR is high and the ER is negative, a second test is recommended [35,40].

HER2-receptor is a protein that controls uncontrolled cell proliferation and promotes tumor growth and spread [37]. In total, 15–20% of patients have elevated HER2 levels [41]. In such patients, specific HER2-targeted therapy has a good effect, whereas in low HER2 levels it is ineffective [42,43,44].

Triple-negative breast cancer (TNBC) is a form of cancer that lacks the estrogen, progesterone, and HER2 receptors. TNBC is detected in 15–20% of patients and is characterized by an aggressive course [45]. The main treatment method is chemotherapy, but new therapeutic targets such as VEGF (a protein responsible for vascular growth) and the androgen receptor are being investigated [46,47]. It was found that the tumor spreads faster at high VEGF levels [47]. In addition to classical markers, genes and nucleic acids (for example, BRCA1 and BRCA2), proteins, and circulating tumor cells are studied [48,49]. The BRCA1 and BRCA2 genes are involved in suppressing tumor growth, but their mutations significantly increase the risk of cancer development [50,51].

Recently, the marker CA 15-3, which is used for the early detection of breast cancer, has become important. Normally, the level of CA 15-3 in human blood does not exceed 30 units/mL. In 30–50% of women with breast cancer, this indicator increases significantly. If the CA 15-3 level exceeds 100 units/mL, this may indicate the presence of a disease. This marker is also used to monitor treatment and detect relapses and metastases [52].

The use of tumor markers makes it possible to more accurately diagnose breast cancer and develop personalized treatment regimens. At the same time, research on new biological markers helps to detect the disease in a timely manner and select individual therapy for each patient. Table 2 shows the biomarkers that are currently the objects of research.

The study of various types of breast cancer biomarkers, including genetic mutations (BRCA1, TP53, and PTEN), protein markers (ER, PR, HER2, and CA 15-3), and microRNAs, plays a key role in diagnosis, prognosis, and therapy selection. Understanding these biomarkers not only provides deeper insights into the molecular mechanisms of tumor growth but also enhances early detection methods. Modern approaches to biomarker analysis focus on increasing sensitivity and specificity, which is particularly important for personalized oncology. In this regard, biosensor technologies are actively evolving, enabling rapid biomolecule detection and opening new possibilities for effective breast cancer diagnostics.

## 3. Application of Biosensors for Breast Cancer Diagnostics

Recently, more and more attention has been paid to the use of biosensors for detecting the biomarkers of tumor cells. Biosensors are able to analyze various signals and detect diseases at an early stage, which allows you to start treatment in a timely manner. Thanks to these devices, patients can monitor their health without relying solely on doctors. Health information is presented in a convenient format, which reduces the complexity of diagnosis [63].

### 3.1. Biosensors for Breast Cancer Diagnostics

Biosensors are analytical devices that convert biological reactions into a measured signal. An ideal biosensor should have high specificity, be resistant to physical parameters (for example, pH and temperature), and be suitable for repeated use [64].

**Figure 4:**
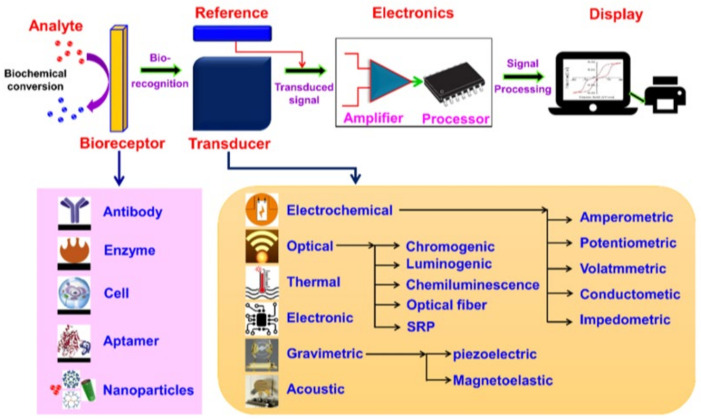
Biosensor components and their characteristics [66].

The structure of the biosensor includes several key components (Figure 4): an analytical material, a bioreceptor, a transducer, and an indicator display. A display connected to electronic or processor-based signal processing systems is used to display the results. Such devices are developed on the basis of various principles of biosensor operation and are designed to detect specific biomarkers. The converter registers data and converts them into an electrical signal, which is then interpreted and presented in a form that is understandable to the user [65].

Biosensors have important characteristics such as selectivity, sensitivity, stability, reproducibility, linearity, and affordable cost, which make them applicable in many areas [67]:

- Selectivity is the ability of an analytical method to determine a specific target component in a complex mixture without the influence of other substances. This quality distinguishes biosensors from other methods, since they allow determining the desired substance without the preliminary separation of the sample [68].
- Biosensor sensitivity is defined as the response signal corresponding to each concentration unit of the target sample [69].
- The stability of a biosensor is its ability to maintain its functionality and accuracy over a long period of time, including the shelf life, the possibility of repeated use, and the ability to work continuously [70].
- Reproducibility—the ability of the biosensor to provide stable and accurate results under the same conditions for a long time. This property is often tested in commercial biosensors and requires periodic calibration to maintain stable results [71].
- Linearity—the ability of the biosensor to provide a proportional and stable response to changes in the input parameter (for example, the concentration of biomolecules). In other words, linearity characterizes the dependence of sensor parameters on the input parameter in the form of a straight line [72].

These characteristics play an important role in evaluating the effectiveness and reliability of the biosensor, as they allow you to predict results and make accurate measurements.

**Figure 5:**
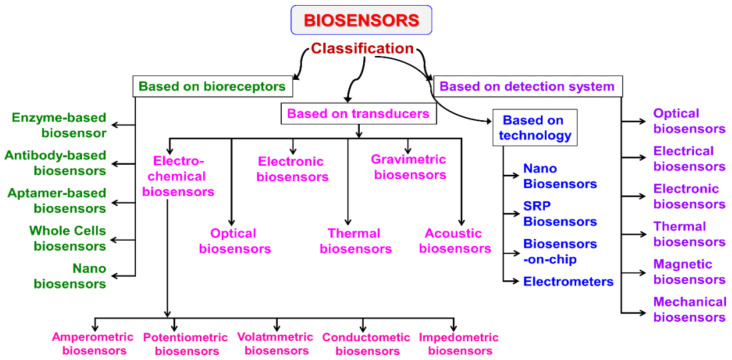
Classification of biosensors [66].

Biosensors are devices designed to detect certain biological or chemical compounds. They are classified according to their structure, operating principle, and technologies used (Figure 5).

This scheme represents the classification of biosensors according to various criteria: the type of bioreceptors, the type of transponders used, and the principle of signal detection. Special attention is paid to electrochemical biosensors, including amperometric, potentiometric, voltammetric and other detection methods, which are of key importance for the development of highly sensitive biomarker analysis systems.

First of all, there are biosensors based on bioreceptors. These include enzymes, antibodies, aptamers, living cells, and nanotechnology-based biosensors. Secondly, biosensors based on transducers are used. They convert the signal into physical, chemical, or electrical parameters. These include electrochemical (amperometric, potentiometric, conductometric, etc.), optical, electronic, thermal, and acoustic biosensors. Each of these types is tailored to a specific application. Third, we can distinguish devices classified by technology: nanobiosensors, surface plasmon resonance biosensors, on-chip biosensors, and electrometers. Also, according to detection methods, a distinction is made, and optical, electrical, thermal, magnetic, and mechanical biosensors are distinguished by detection methods.

In order to further characterize the stability of the electrochemical biosensors, the storage stability is tested. And in various types of biosensors, a difference in the operational stability and shelf-life is shown. For instance, Hao Ji and other scientists published a paper where InSe-FETs were stored in the atmosphere and liquid for five days, respectively. The peak intensity of the InSe immersed in the 0.1 PBS solution declined by 7.43% after five days, while that of the InSe exposed to the atmosphere declined by 35.68%. The decrease in the PL peak intensity is probably due to the formation of InSe oxides. With a standard error of less than 8.78%, the suggested biosensor can detect the antigen CA125 over an extremely wide range, from 0.01 to 1000 units/mL. Clinical sample detection has demonstrated that InSe-FET biosensors have a lot of potential for real-world uses, including real-time health monitoring, studying the pathophysiology of major diseases, and early cancer diagnosis and prognosis [73].

Iranian and Turkish scientists published a paper titled “Pathological Validation of Electrochemical Biosensor for Breast Cancer Detection using Enhanced Molybdenum Polyataurine Nanofilm”. The molybdenum trioxide/polytaurine nanofilm was synthesized by electrochemical method for HER2 monitoring in a breast cancer patient. The results of HER2 biomarker detection in human blood were compared with the immunohistochemical IHC method. Noting that this sensor is designed for accurate, rapid, and specific detection, it has a detection limit (0.000001 ng/mL) and a linear dynamic range (0.1–0.000001 ng/mL). The sensitivity, simplicity, and reproducibility of electrochemical biosensors were improved [74].

Xiao Wang et al. developed an electrochemical immunosensor for the quantitative detection of RPE biomarkers. Under the sensing signal of HMSNs-Cu^2+^@HA, the structure of HB was degraded in an acidic medium, and the released Cu^2+^ ion activated the MMoO_4_ (M = Co, Ni)-based substrate. Using the CoMoO_4_ substrate as an electrode, the detection range was 0.01 pg/mL to 40 ng/mL, and the detection limit was 0.0035 pg/mL. The results showed excellent selectivity, reproducibility, and stability of the electrochemical sensor. Thus, the biosensor incorporated a novel method for the early diagnosis of CEA [75].

In summary, it is shown that the operational stability and shelf-life of various bio-sensor types depends on different factors, from the components of the modified objects to the temperature and condition of storage [75].

### 3.2. Classification of Biosensors

Biosensors are classified based on the type of biological receptor, signal transduction method, and technological features. Depending on the receptor type, they may contain biomolecules that selectively bind to target compounds. Based on the transducer principle, these devices utilize physicochemical changes to register signals. Modern developments include technologies that enhance sensitivity and accuracy through advanced materials and miniaturization. This classification helps identify the most suitable biosensor systems for various applications, including medicine, biotechnology, and environmental monitoring.

#### 3.2.1. Biosensors Based on Bioreceptors

- Enzyme biosensors are devices that use an enzyme as a biological receptor. They have high catalytic activity and selectivity, accelerating biochemical reactions and providing accurate and specific analyte determination [76].
- Antibody-based biosensors are devices that use antibodies or antigens as a biological element. Such biosensors are usually referred to as “immunosensors” [77].
- Aptameric biosensors are devices in which the biological element is aptamers–synthetic oligonucleotides with high selectivity and affinity [78].
- Whole cell-based biosensors are devices that use living cells to detect target substances, providing a natural and complex interaction with the analyzed compounds [79].
- Nanobiosensors are devices that use nanostructures to improve the interaction between a biological element and a transducer [80].

#### 3.2.2. Biosensors Based on Transducers

- Electrochemical biosensors are devices that use electrochemical processes to detect substances and consist of three electrodes (working, auxiliary, and reference) [28].
- Amperometric biosensors are electrochemical biosensors that measure the current by amperometry at a given potential [81].
- Potentiometric biosensors are devices that measure the potential difference between the working and reference electrodes at a minimum current (~10^−15^ A) [82].
- Voltammetric biosensors are devices that measure current changes during the redox reactions of electroactive substances on an electrode [83].
- Optical biosensors are devices that measure light as a converted signal. They are based on optical diffraction or electrochemiluminescence [84].
- Electronic biosensors are devices that work by converting biochemical changes into electrical signals [85].
- Thermal biosensors are devices that measure the thermal energy released or absorbed as a result of a biochemical reaction [86].
- Gravimetric biosensors are devices that generate a signal based on changes in mass [87].
- Acoustic biosensors are devices that use piezoelectric materials to generate and detect acoustic waves [88].

#### 3.2.3. Technological Classification

- Surface plasmon resonance-based biosensors are sensors that use the optical measurements of the changes in the refractive index during the interaction of an analyte with a biomolecular element [89].
- Biosensors on a chip are devices that combine biological sensing elements with microfluidic technologies, allowing the accurate determination of biological and chemical components in various samples [90].
- Electrometers are high-precision devices used to measure electric charge and voltage [91].

The classification of biosensors helps systematize modern biomolecule detection technologies by categorizing them based on bioreceptors, transducers, and technological features. The advancement of these devices enhances the accuracy, sensitivity, and speed of analysis, making them indispensable in medicine, biotechnology, and environmental monitoring. Current trends focus on miniaturization, automation, and integration with digital platforms, expanding their potential applications in diagnostics and scientific research.

### 3.3. Analysis of Electrochemical Biosensors

Bioreceptors can be enzymes, cells, aptamers, DNA, RNA, or antibodies with high selectivity and the ability to specifically recognize the analyte. This section details the use of electrochemical sensors for the detection of breast cancer biomarkers using electrochemical methods based on different bioreceptors. The sensors can be categorized primarily into three main types: sensors using voltammetry, electrochemical impedance spectroscopy (EIS), and amperometry techniques [92].

#### 3.3.1. Biosensors Based on Electrochemical Impedance Spectroscopy (EIS)

Impedance biosensors are created using an alternating voltage or low-amplitude current at wide frequencies, monitoring changes in impedance at the electrode interface [90]. Because of their obvious advantages, EIS-based electrochemical sensors are ideal for high sensitivity and unlabeled (markerless) analysis (e.g., wide measurement range, low detection threshold, fast response time, easy preparation, and stability) [93].

The studies report the detection of biomarkers such as HER1 (cerbB1), EGFR, HER-2 (neu/cerbB2), BRCA1, miRNA-34a, miRNA-155, HER-3, and p53 by the EIS method [91]. The EIS method measures how the resistance of a system changes when biological molecules (such as proteins or aptamers) attach to the surface of an electrode. The electrochemical cell is subjected to an alternating voltage over a wide range of frequencies to determine its electrochemical impedance. This impedance describes the ability of the system to resist the flow of electric current. The impedance spectrum is then recorded.

The EIS method uses electroactive compounds such as Fe(CN)F^3-/^Fe(CN).^-^, which are oxidized and reduced at the working electrode. This method allows us to quantify the increase in charge transfer resistance with the gradual formation of a bioconductor layer on the electrode surface [94].

#### 3.3.2. Voltammetric Biosensors

Electrochemical sensors have been developed by combining voltammetry and immunoassay and allow current measurement depending on the potential used. This method has the advantage of low non-specific adsorption and less background interference. To increase the sensitivity and selectivity of the working electrode, different methods apply a certain voltage (e.g., step and linear gradient) [95].

The voltammetric method is based on measuring the change in current and voltage at the working electrodes. In biosensorics, the following methods are used:

- Cyclic voltammetry (CV);
- Linear voltammetry (LV) [96];
- Differential pulse voltammetry (DPV);
- Square-wave voltammetry (SWV) [97].

#### 3.3.3. Differential Pulse Voltammetry (DPV)

The DPV method utilizes non-continuous voltage pulses to reduce the charge current, demonstrate high sensitivity, and provide a low detection threshold (LOD). The current is measured only before a potential change occurs [98].

In the DPV method, the current at two points of each pulse is measured with respect to the potential. The electrode is given a base potential that increases with a constant increase between pulses when no oxidative or reductive process is occurring in the system. To record the differential current, the current value is taken at the points before and after the pulse.

The main advantage of the DPV method is its high signal-to-noise ratio, which makes it widely used in biosensorics. This method is often used in combination with electroactive substances to further amplify analytical signals [99]. It is suitable for determining the biomarkers miRNA-155, HER1 (cerbB1/EGFR), HER2, CA15-3, BRCA1, CEA, and VEGFR2.

#### 3.3.4. Square-Wave Voltammetry (SWV)

SWV is an electrochemical method in which the resultant current is measured relative to the applied voltage. In this method, the potential of the working electrode is changed using a sequence of forward and reverse pulses. Here, the quadratic amplitude defines the forward step, and the quadratic magnification defines the reverse step. The SWV method has high sensitivity and the ability to give rapid results, making it more efficient than other electrochemical methods. In addition, the analytical time based on SWV is shorter than the DPV method, which reduces the damage to biological substances such as cells in real samples [100].

**Figure 6:**
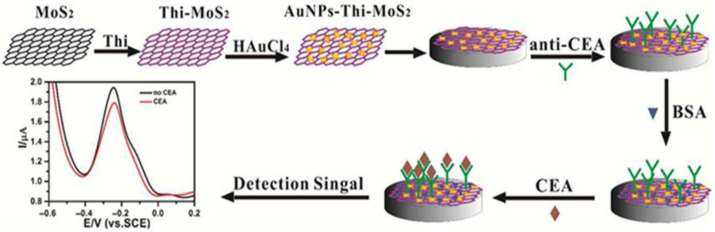
The scheme of modification of electrodes on the basis of MOS_2_ [102].

CEA is a serum glycoprotein that is a marker for breast cancer. SWV was used to detect the CEA marker of breast cancer using the BSA/antiCEA/AuNPs-Thi-mos_2_/GCE sensor. The sensor platform used gold nanoparticles (AuNPs) decorated with MoS_2_ and Thi (Figure 6), which increased the sensitivity. When the biomarker CEA was detected, a decrease in current was observed at the point with higher Thi. The detection limit of this sensor was 0.52 pg/mL, and the detection range was from 1.0 pg/mL to 10.0 ng/mL [101].

The illustration demonstrates the process of functionalization of molybdenum disulfide (MOS_2_) using thiol groups and subsequent modification with gold nanoparticles. This approach makes it possible to increase the electrical conductivity of the material and ensure the selective immobilization of antibodies against cancer antigens (CEAs), which is critical for the sensitive and specific detection of this marker using an electrochemical response.

#### 3.3.5. Linear Voltammetry (LV)

The LV method measures current by scanning at a constant speed. This method is used to determine the electrochemical properties of the substrate (oxidation/reduction), analyze the reaction intermediates, identify the anode limits, and evaluate the reversibility of the reaction. In comparison with other electrochemical methods, LV demonstrates high efficiency in detecting a variety of analytical substances [103].

#### 3.3.6. Cyclic Voltammetry (CV)

The CV method is used in biosensors for the early diagnosis of breast cancer biomarkers. CV is a voltammetric method that studies the electrochemical properties of electrically conductive materials. When the working electrode potential changes in the forward and reverse directions, the corresponding current is measured [104].

The antigen–antibody binding elements HER1, HER2, and HER3 were determined using the CV method. In addition, biomarkers such as microRNA-21, microRNA-155, HER2, IL 6, CA 15-3, and HER-2 ECD were studied using the SWV and CV methods.

## 4. Overview of the Structure of Electrochemical Biosensors for the Detection of HER-2

In our opinion, the versatility of biosensors allows them to be widely used in science and practice. For example, electrochemical biosensors are the most common type among all biosensors due to their efficient operating principle and portable size. More than 70% of all electrochemical biosensors are used in devices for determining blood glucose levels [105].

Therefore, we showed interest in electrochemical biosensors and conducted a brief review of the research in this area (Table 3).

Table 3 provides a comparative review of electrochemical biosensors designed for the detection of HER2 and related biomarkers. By analyzing development methods and key characteristics, it is possible to identify both the strengths and weaknesses of various approaches.

One of the most sensitive sensors is CS/(Ru(BPY)_3_)^2+^/RGO/GCE [110], fabricated using electrodeposition, achieving a detection limit of 1 femtomole (1 fM). The use of reduced graphene oxide (rGO) enhances signal stability but decreases selectivity, which remains a limitation. In nanomaterial composites such as AuNP/DPB [107], sensitivity reaches 0.037 pg/mL, but the complexity of synthesis and the limited stability of aptamers require further optimization.

Molecularly imprinted polymer-based materials (MIP/AuSPE) [108] provide a sensitivity of 1.6 ng/mL, offering advantages in simplicity and cost-effectiveness. However, they fall short compared to nanostructured electrodes. On the other hand, biosensors incorporating metallic nanoparticles [106,115] (AuNP (SPGE) and graphite electrodes with Rh and rGO) achieve a balance between high sensitivity and stability, although long-term reproducibility remains a challenge.

Metal–organic framework (MOF) composites such as ZIF-67@Fc/AMNF [112] and AMNFs@ZIF-67 [114] demonstrate remarkable sensitivity, reaching 4.853 fg/mL. However, their low conductivity limits electrochemical performance, necessitating functionalization with conductive polymers.

Electrochemical DNA immobilization (Polycytosine DNA) provides high selectivity, but the stability of such biosensors remains an unresolved issue. In the case of Fe_3_O_4_@TMU-21 [122], encapsulation enables a low detection limit of 0.3 pg/mL; however, electrode instability with changing pH conditions calls for buffer optimization.

One of the most promising strategies involves the use of hybrid nanomaterials, such as graphene–metal oxide structures, as well as improvements in electrode functionalization methods. The integration of biosensors into multiplexed platforms will enhance diagnostic accuracy and accelerate clinical implementation.

## 5. Factors That Prevent the Determination of the HER2 Biomarker

HER2 (human epidermal growth factor receptor 2) is a specific protein located on the cell surface. The HER2 gene encoding this receptor is located on chromosome 17q21. HER2 is a protein weighing approximately 185 kDa, consisting of 1255 amino acids [126]. It belongs to the family of tyrosine kinase receptors and plays a key role in the transmission of signals from the external environment into the cell.

**Figure 7:**
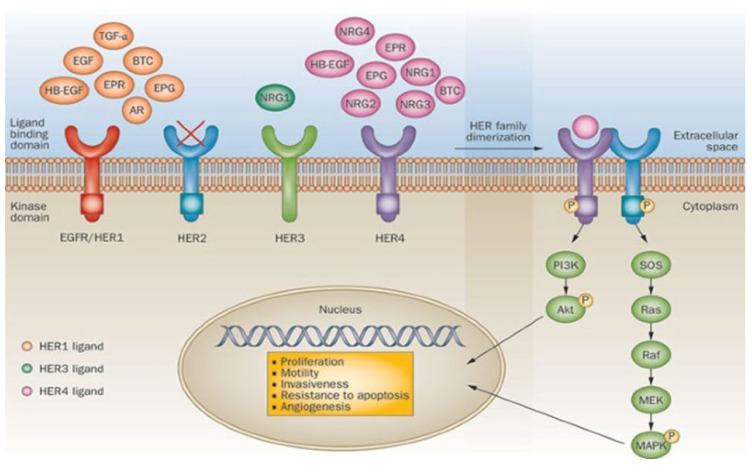
Molecular mechanisms of signal transmission by HER receptors [128].

The process of activating the HER2 receptor triggers a chain of biological processes leading to cell growth, division, or survival. This signaling pathway regulates important processes in the cell and consists of several stages [127], which can be represented as a diagram (Figure 7).

This image illustrates the functioning of the HER receptor family, their interaction with ligands, and involvement in the processes of cell proliferation, migration, and angiogenesis. Special attention is paid to the HER2 receptor, which plays a key role in the pathogenesis of breast cancer and is an important target for the diagnosis and therapy of the disease.

The HER2 receptor plays a crucial role in cell signaling by undergoing activation processes that trigger intracellular pathways responsible for cell growth, division, and survival.

1. Dimerization: When the HER2 receptor is activated, it binds to other receptors (such as HER4) or to itself. This process is called dimerization and activates the interior of the receptor.
2. Transphosphorylation: Once activated, the receptors undergo special chemical changes to their internals (phosphorylate them). These changes serve as the starting point for signaling to other molecules.
3. Signal transduction pathways: The signal from the HER2 receptor travels through specialized pathways within the cell, triggering cell growth, division, or survival.

In breast cancer, the HER2 pathway is often overactivated, leading to uncontrolled cell growth and division. This highlights the important role of the HER2 receptor in cancer.

The HER2 signaling pathway includes several levels:

- External level: The HER2 receptor receives a signal outside the cell.
- Internal level: The signal is transmitted inside the cell.
- Nuclear level: A signal reaches the nucleus, altering gene activity and cell properties.

Impaired or excessive HER2 activity can contribute to the onset and spread of cancer. Therefore, HER2 is a key target for cancer research and therapy.

In general, HER2 is a protein that is often detected in breast cancer. As a result of the destruction of the outer part of the membrane, it appears in a soluble form in serum and plasma, which allows you to measure the level of HER2 to monitor the progression of the disease and the effectiveness of treatment.

Currently, serum samples are often used to measure HER2 levels, as this method is approved by the FDA, and the threshold concentration is set at 15 ng/mL. In healthy people, the concentration of HER2 in the serum is 5–10 ng/mL, and in HER2-positive patients at risk of cancer, it can reach several hundred ng/mL [129]. At the same time, the serum contains no fibrinogen [130]. Fibrinogen is a protein of the hemostatic system synthesized by hepatocytes and converted to fibrin by the action of thrombin. Due to its ability to participate in blood clotting and dense matrix formation, fibrinogen can interfere with the determination of HER2, since its associated products are detected in significant quantities in various solid tumors [131].

When using biosensors for HER2 detection, it is important to take into account possible analytical interference and its sources [132] in order to minimize their impact on the results obtained. Table 4 shows the main analytical environments, potential interferences, and methods for their elimination:

Detecting and minimizing interference plays a key role in improving the efficiency of biosensors. The absence or minimization of interference improves the selectivity, sensitivity, and reproducibility of biosensors, which contributes to reliable and linear results [133].

## 6. Discussion

Modern breast cancer diagnostic methods include a wide range of technologies, ranging from traditional imaging techniques such as mammography and ultrasound (ultrasound) to molecular and biosensor approaches focused on the detection of specific biomarkers. Despite their diagnostic value, traditional methods have a number of limitations, including high costs, false-positive and false-negative results, and limited applicability for the early detection of the disease.

An analysis of existing diagnostic methods (see Table 1) shows that mammography remains the main tool for mass screening, but its effectiveness decreases in women with high breast density. Molecular diagnostic methods such as FISH, IHC, and liquid biopsy are highly specific but require significant costs and complex laboratory infrastructure. At the same time, computed tomography (CT) and positron emission tomography (PET) make it possible to assess the stage of the disease and the spread of metastases in detail, but they are not always applicable as primary diagnostic tools.

The study of breast cancer biomarkers reveals promising targets for diagnosis and therapy. Genetic markers such as BRCA1, BRCA2, TP53, and PIK3CA can predict predisposition to the disease and personalize treatment. Proteomic biomarkers, including CA15-3, CA27-29, and HER2, are used to monitor tumors and determine their biological behavior. In recent years, active research has been conducted on the role of microRNAs such as miR-146a and miR-1290 in the progression of the disease.

The most promising area of diagnostics is the development of biosensors that provide the rapid detection of biomarkers with high sensitivity and selectivity. Biosensors based on enzymes, antibodies, and aptamers demonstrate the possibility of the rapid and non-invasive detection of cancer cells, which is especially important for screening in the early stages of the disease. Electrochemical and optical biosensors are already showing promising results in clinical trials, enabling the accurate monitoring of tumor dynamics.

When developing biosensors for the diagnosis of breast cancer, it is important to take into account the influence of interfering agents that can reduce the accuracy of the analysis. Biological fluids such as blood and saliva contain various endogenous compounds (e.g., albumin, glucose, and urea) capable of causing non-specific binding or masking target biomarkers. This can lead to false-positive or false-negative results.

An additional problem is external pollutants and variability in the composition of samples in different patients. To minimize these effects, selective sensor surfaces, the use of blocking agents, and signal processing algorithms that increase detection accuracy are being developed.

## 7. Conclusions

Breast cancer remains one of the leading causes of morbidity and mortality among women worldwide, which requires the development of more effective diagnostic approaches. Summing up the work performed, the following conclusions can be drawn:

- Modern breast cancer diagnostic methods are highly informative but have significant limitations in sensitivity, specificity, and accessibility.
- The use of biomarkers allows you to personalize the diagnosis and predict the effectiveness of treatment, which is an important step toward individualized medicine.
- Biosensors represent a promising alternative to traditional diagnostic methods, providing high-speed analysis, minimal invasiveness, and accessibility.
- Further research should be aimed at modifying biosensor technologies, improving their properties for simplification and ease of use, as well as adapting them to mass population screening.

Thus, the introduction of new technologies such as biosensors and molecular diagnostic methods will increase the accuracy of breast cancer detection and improve disease prediction while reducing the financial burden on the healthcare system.

## Figures and Tables

**Table 1:** Characteristics and disadvantages of breast cancer diagnostic methods.

№	Method	Features	Disadvantages	Ref.
1	Mammography	Allows the detection of various breast pathologies at early stages and assesses the risk of their development.	Identifies only suspicious areas; additional methods are required for diagnosis clarification.	[15]
2	Ultrasound (US)	Used to evaluate palpable masses, nipple changes, and lactation-related pain.	Difficult to distinguish between malignant and benign tumors; biopsy is required.	[16]
3	Magnetic Resonance Imaging (MRI)	High-resolution imaging of soft tissues, allows the determination of the lesion area and the extent of spread.	Too expensive for routine use in mass screening.	[17]
4	Positron Emission Tomography (PET)	Used for disease staging and metastasis detection.	May give false-positive results in inflammatory processes, infections, or fibrotic changes.	[18]
5	Computed Tomography (CT)	Widely used to detect metastases in organs (lungs, liver, and bones) in advanced stages of breast cancer.	Uses X-ray radiation, limiting its application in mass screening, especially in women with dense breast tissue.	[19]
6	Single Photon Emission Computed Tomography (SPECT)	Assesses functional tissue characteristics (blood supply and metabolism), which is effective in determining tumor activity.	Difficulty in precise tumor localization and the detection of small formations.	[20]
7	Biopsy	Depending on the type of biopsy, minimal invasiveness is possible. Effective in detecting circulating tumor cells and DNA.	Provides only a momentary snapshot of the tumor state, which may not reflect its heterogeneity and dynamic changes during treatment.	[21,22]

**Table 2:** Types and characteristics of breast cancer biomarkers.

№	Biomarker Name	Description	Ref.
1	TP53 (p53)	One of the most frequently mutated genes in breast cancer is TP53 (p53). Although this gene is mutated in approximately 30–35% of all cases, in triple-negative breast cancer (lack of ER, PR, and HER2 receptors), the mutation rate reaches 80%. Thus, mutated p53 plays a crucial role as a biomarker and therapeutic target in this type of cancer.	[53]
2	BRCA1/BRCA2	Pathogenic or potentially pathogenic mutations in the BRCA1 gene increase the predisposition to triple-negative breast cancer. Meanwhile, BRCA2 mutations are more often associated with estrogen receptor-positive tumors.	[54]
3	PTEN	Loss of PTEN or decreased expression can affect patient prognosis. Recent studies indicate that low PTEN levels lead to unfavorable outcomes in HR+/HER2− and HER2+ tumors.	[55]
4	CHEK2	Carriers of the CHEK2 × 1100delC allele have an increased risk of developing breast cancer, but this risk decreases with age. Studies show that such tumors are more often estrogen receptor-positive, although the influence of progesterone receptors and HER2 remains unclear.	[56]
5	PALB2	One of the key genes whose mutations are associated with metastatic breast cancer.	[57]
6	BRIP1	A tumor suppressor gene that ensures genetic stability through DNA repair. However, mutations or increased BRIP1 expression can directly contribute to breast cancer development.	[58]
7	CDH1	Depending on the type of biopsy, minimal invasiveness is possible. Effective in detecting circulating tumor cells and DNA.	[59]
8	PIK3CA	E-cadherin protein, responsible for cell adhesion. Loss of its function is associated with tumor metastasis, as it facilitates cell movement and invasion into surrounding tissues.	[60]
9	MicroRNAs	Elevated levels of mir-3662, mir-146a, and mir-1290 in exosomes of breast cancer patients correlate with disease progression and lymph node metastases.	[61]
10	Upa/PAI-1	High levels of Upa-PAI-1 complexes in tumors are associated with reduced survival in early-stage breast cancer patients and poorer therapy response. However, the exact mechanism of their influence on tumor development remains unclear.	[62]

**Table 3:** Brief overview of the structure and properties of electrochemical biosensors.

Sensor (Electrode Surface Composition)	Research Method	Synthesis Method	Analyte	Detection Limit	Linear Range	Advantages	Disadvantages	Ref.
AuNP (SPGE)	Amperometry	Nanomaterial modification	HER2, HER1	HER2: 0.95 ng/mL, HER1: 1.06 ng/mL	5–200 ng/mL	High sensitivity, early detection capability, and disease progression monitoring	May require significant time	[106]
AuNP/DPB nano- composite	Voltammetry	Nanomaterial modification	HER2	0.037 pg/mL	0.1 pg/mL—100 ng/mL	High sensitivity, selectivity, and suitability for clinical tumor cell analysis	Complex bioconjugate synthesis and limited aptamer biosensor stability (sensitivity to storage conditions)	[107]
MIP/AuSPE	EIS, CV	Electropolymerization of solution	HER2-ECD	1.6 ng/mL	10–70 ng/mL	Simplicity, ease of use, cost-effectiveness, and ability for selective analysis	Possible interference from other molecules and need for additional clinical trials	[108]
SPCE/AuNP	LV	Nanomaterial modification	HER2-ECD	0.16 ng/mL	7.5–50 ng/mL	Stable antibody immobilization ensures high sensitivity, and a strong analytical signal obtained in a short time	Limited long-term stability and reproducibility	[109]
CS/[Ru(BPY)_3_]^2+^/RGO/GCE	DPV, SWV	Electrodeposition	HER-2 (AB60866) and HER-2 antibody (AB214275)	1 fM	1 fM–1 nM	Use of reduced graphene oxide (rGO) as a substrate enhances signal stability and sensitivity	Low signal stability and reduced selectivity	[110]
GNR@Pd—Apt—HRP	LV	Electrodeposition	HER2 ECD	4.4 ng/mL	15–100 ng/mL	High selectivity	Long analysis time	[111]
ZIF-67@Fc/ AMNF, ZIF-90@MB	Amperometry	Nanomaterial modification	HER2	55 fg/mL	0.5–1000 pg/mL	High accuracy, sensitivity, and reproducibility	Complex synthesis of functionalized MOFs (ZIF-67 and ZIF-90)	[112]
(PEDOT) and biodegradable peptide hydrogel	DPV	Self-assembly of peptide hydrogel on electrode surface	HER2	45 pg/mL	0.1 ng/mL–1.0 µg/mL	Ensures high activity of immobilized biomolecules, and hydrophilicity prevents unwanted adsorption	Limited stability	[113]
AMNFs@ZIF-67	SWV	Adsorption	HER2	4.853 fg/mL	0–1000 pg/mL	High sensitivity and stability	Low conductivity of MOFs (metal–organic framework materials)	[114]
Graphite electrode (GE) with reduced graphene oxide (rGO) and rhodium (Rh) nanoparticles	EIS, DPV, CV	Nanomaterial modification	HER2-ECD	0.667 ng/mL	10.0–500.0 ng/mL	Wide dynamic range, high sensitivity, selectivity, stability, and reproducibility, and low cost	Significant decrease in peak current observed in the study	[115]
Cu-MOF and Cu_2_ZnSnS_4_ NPs/Pt/gC_3_N_4_	CV	Layer-by-layer nanomaterial deposition	HER2	0.01 fg/mL	0.01–1.00 pg/mL	High sensitivity and selectivity and wide linear range	Complex synthesis process	[116]
Halloysite nanotubes/Pd (HNT/C@Pd NPs)	EIS	Hydrothermal method	HER2	8 pg/mL	0.03–9 ng/mL	High sensitivity and selectivity, and stability	Possible lack of linear range	[117]
LSG-AuNS	DPV	Laser engraving and electrolytic deposition	HER2-ECD	1 pg/mL	0.1–10 ng/mL	High sensitivity and fast response time	Stability evaluation required	[118]
ZIF-8/2D Co-MOF	Potentiometry	In situ electrochemical deposition	HER2/ER	3.8 fg/mL, 6.8 fg/mL	0–15 pg/mL	High conductivity, large surface area, and simple structural assembly	Stability and reproducibility not fully studied	[119]
Polycytosine DNA (dc20)	Amperometry	Nanoparticle immobilization	HER2	0.5 pg/mL	1 pg/mL–1 ng/mL	High selectivity and efficiency	Biosensor stability requires further study	[120]
BiOBr_0.8_I_0.2_/CoS^x^	Voltammetry	Hydrothermal method	HER2	1.06 pg/mL	0.005–15 ng/mL	High cathodic signal and high selectivity and stability	Optimal conditions required for low concentration detection	[121]
Fe_3_O_4_@TMU-21	Amperometry	Encapsulation	HER2	0.3 pg/mL	0–100 ng/mL	High sensitivity and selectivity	Electrode properties’ instability depending on pH conditions	[122]
2D functionalized graphene oxide (FGO)	Potentiometry	In situ electrochemical oxidation	HER2	0.59 ng/mL	0.5–25 ng/mL	High conductivity and surface area due to FGO and enhanced electrochemical signal	Analysis time for analytes not determined	[123]
Au@Ag NR	DPV	In situ growth	HER2	16.7 fg/mL	50 fg/mL–100 pg/mL	Accelerated electron transfer and enhanced current signal	Stability and reproducibility not fully studied	[124]
N-SQDs/GS	Voltammetry	Electrodeposition	HER2	4.8 pg/mL	0.1–1 ng/mL	High selectivity, sensitivity, and stability	Long signal acquisition time	[125]

**Table 4:** Factors that hinder the determination of HER2, and methods for their elimination.

Sample Type	Interfering Agents	Interference Mechanism	Elimination Methods
Blood serum	Proteins (albumin and globulins)	Compete with HER2-binding antibodies, causing non-specific reactions	- Use of blocking agents (BSA and casein) - Removal of proteins by ultrafiltration or precipitation
	Lipids	Disrupt the binding of HER2 to antibodies and affect optical measurements	- Centrifugation - Use of lipid-breaking enzymes (lipase)
	Hemoglobin (the result of hemolysis)	Distorts the results of optical and electrochemical methods	- Exclusion of highly hemolyzed samples - Pre-filtration
	Bilirubin	Performs optical detection of HER2	- Dilution of samples - Use of spectral correction methods
Blood plasma	Anticoagulants (heparin and EDTA)	Inhibit antibody binding or affect measurements	- Selection of reagents compatible with anticoagulants - Optimization of buffer conditions
	Proteins and lipids	Compete with HER2-binding antibodies, causing non-specific reactions	- Use of blocking agents (BSA and casein) - Removal of proteins by ultrafiltration or deposition
Tissue samples (biopsy)	Cell residues and DNA	Interfere with HER2 visualization or create background fluorescence	- Pretreatment (homogenization and centrifugation) - Purification using specific antibodies
	Lipids and cell residues	Prevent antibodies from binding to the target HER2 protein	- Tissue washing - Use of detergents
Urine	Low levels of HER2	Make detection difficult due to the composition of the liquid	- Concentration of samples - Use of highly sensitive methods (for example, ELISA or PCR)
	Salts and urea	Inhibit the antigen–antibody interaction	- Dilution of samples - Use of special buffers
Saliva	Proteases	Destroy the HER2 protein, causing false results	- Addition of protease inhibitors—immediate processing of samples
	Low levels of HER2	Make detection difficult due to low concentrations	- Concentration of samples - Use of sensitive methods
